# Supplementary material for: Transfer of celiac disease-associated immunogenic gluten peptides in breast milk: variability in kinetics of secretion
Source: Front Immunol. 2024 Jul 5;15:1405344. doi: 10.3389/fimmu.2024.1405344 (PMC11257844; doi:10.3389/fimmu.2024.1405344)
Supplement: Supplementary file 1 [file DataSheet_1.pdf]

A

	DAY -2	DAY -1	DAY 0	DAY 1	DAY 2	DAY 3	DAY 4
**Breakfast**	**GCD** **Toast**	**GCD** **Toast**	**GCD** **Toast**	GFD Coffee / Fruit/Greek natural yogurt	GFD Coffee / Fruit/Greek natural yogurt	GFD Coffee / Fruit/Greek natural yogurt	GFD Coffee / Fruit/Greek natural yogurt
**Lunch**	**GCD** **Pasta/Bread or Croutons/ lasagna**	**GCD** **Pasta/Bread or Croutons/ lasagna**	GFD ▪ Ratatouille ▪ Fruit/Greek natural yogurt	GFD ▪ Lentils ▪ Fruit/Greek natural yogurt	GFD ▪ Grilled tuna ▪ Dressing with fish eggs ▪ Fruit/Greek natural yogurt	GFD ▪ Baked chicken ▪ Bonito in marinade ▪ Fruit/Greek natural yogurt	
**Dinner**	**GCD** **Pizza/Sádwich/Wheat pancakes**	**GCD** **Pizza/Sádwich/Wheat pancakes**	GFD ▪ Omelette ▪ Salad ▪ Fruit/Greek natural yogurt	GFD ▪ Octopus marinade ▪ Chicken ▪ Fruit/Greek natural yogurt	GFD ▪ Garlic chicken ▪ Fruit/Greek natural yogurt	GFD ▪ Grilled mushrooms ▪ Fruit/Greek natural yogurt	

B

DAY -2	DAY -1	DAY 0	DAY 1	DAY 2	DAY 3	DAY 4
		**Collection of breast milk and urine samples:** **1º) After breakfast** **2º) 3 hours after the 1st sample** **3º) 6 hours after the 1st sample**	**Collection of breast milk and urine samples:** **1 sample at 24 hours from the 1st sample**	**Collection of breast milk and urine samples:** **1 sample at 48 hours from the 1st sample**	**Collection of breast milk and urine samples:** **1 sample at 72 hours from the 1st sample**	**Collection of breast milk and urine samples:** **1 sample at 96 hours from the 1st sample**

Figure 1. (A) Menu planning. (B) Sample collection planning. GCD, gluten-containing diet; GFD, gluten-free diet.
